# Supplementary material for: International medical graduates’ experiences of clinical competency assessment in postgraduate and licensing examinations: A scoping review
Source: PLoS One. 2026 Apr 30;21(4):e0338282. doi: 10.1371/journal.pone.0338282 (PMC13132449; doi:10.1371/journal.pone.0338282)
Supplement: S3 Appendix — (DOCX) [file pone.0338282.s003.docx]

S3: Appendix 3: Data Extraction Tool

| **Scoping Review Details** | |
| --- | --- |
| Scoping Review title: | International medical graduates’ experiences of clinical competency assessment in postgraduate and licensing examinations: a scoping review protocol |
| Review objective/s: | The objective of this scoping review is to examine the major concepts in academic and grey literature relating to international medical graduates’ experiences of clinical competency assessment; and to identify the gaps in our knowledge on this topic. |
| Review question/s: | 1. What literature has been published relating to the experiences of international medical graduates undertaking clinical postgraduate and licensing medical examinations? 2. What experiences do international medical graduates describe in relation to clinical postgraduate and licensing medical examinations? 3. What are the gaps in the literature relating to our knowledge and understanding of international medical graduates’ experiences of clinical postgraduate and licensing medical examinations? |
| **Inclusion/Exclusion Criteria** | |
| Population | International medical graduates |
| Concept | Experiences of clinical competency assessment |
| Context | Postgraduate or licensing or credentialing medical assessment designed to measure clinical competence |
| Types of evidence source | - Qualitative or mixed methods studies, ‘grey’ literature such as reports, reviews, theses, letters, book chapters, opinion pieces, and organisational documents - Published between 2009 - 2025. |
| **Evidence source Details and Characteristics** | |
| Citation details (e.g., author/s, date, title, journal, volume, issue, pages) |  |
| Country |  |
| Context |  |
| Participants (details e.g., age/sex and number) |  |
| **Details/Results extracted from source of evidence** (in relation to the concept of the scoping review) | |
| Experiences described by IMGs in relation to assessment |  |
| IMG’s views on their assessments / outcomes |  |
| Data published by postgraduate training bodies relating to IMGs and assessment |  |
| Data published by medical licensing organisations relating to IMGs and assessment |  |
| Recommendations published by postgraduate training bodies relating to IMGs and assessment |  |
| Recommendations published by medical licensing organisations relating to IMGs and assessment |  |
