# Supplementary material for: International medical graduates’ experiences of clinical competency assessment in postgraduate and licensing examinations: A scoping review
Source: PLoS One. 2026 Apr 30;21(4):e0338282. doi: 10.1371/journal.pone.0338282 (PMC13132449; doi:10.1371/journal.pone.0338282)
Supplement: S4 Appendix — (DOCX) [file pone.0338282.s004.docx]

S4: Appendix 4: Description of the Sources

| **Year** | 2009, n=1 (77)  2010, n=5 (47, 50, 51, 54, 57)  2011, n=2 (62, 66)  2012, n= (58, 74, 76, 82, 88)  2013, n=2 (69, 72)  2014, n=2 (67, 84)  2015, n=4 (71, 73, 83, 86)  2016, n=3 (56, 78, 79)  2018, n=1 (52)  2019, n=1 (87)  2020, n=8 (53, 60, 63, 64, 65, 68, 80, 89)  2021, n=1; (61)  2022, n=1 (70)  2023, n=2 (49, 59)  2024, n=4 (3, 20, 75, 85)  2025, n=2 (55, 81) |
| --- | --- |
| **Country of publication** | **UK**, n=14 (35,47,56,63,66,69,70,73,75,76,78,79,83,88)  **Australia**, n = 13 (3, 20, 50, 54, 55, 58, 64, 71, 81, 84, 88, 89)  **Canada**, n=5 (53,57,59,60,72)  **USA,** n=3 (62,65,74)  **Netherlands**, n=2 (49,51)  **Chile**, n=1 (86)  **Finland**, n=1 (67)  **Germany**, n=1 (80)  **Sweden**, n=1 (53)  **Australia and New Zealand**, n=2 (77,82)  **Multiple**, n=1 (68) |
| **Type of study** | **Qualitative**, n=25 (3,20,49,51-52,56–59,62–66,68,71–73,76,78–80,82-83,91)  **Quantitative**, n=5 (50,69,74,77,88)  **Mixed**, n=13 (35,53, 55,60,61, 67,70,74,75, 81,84,89,90)  **N/A**, n=1 (47) |
| **Examination** | **Licensing examinations**, n=22  **Australian Medical Council (AMC) Clinical Exam**, n=5, (3,20,58,64,84)  **AMC Workplace Based Assessment (WBA) pathway,** n=6 (55,61,71,81,87,90)  **Medical Council of Canada’s (MCC) National Assessment Collaboration (NAC OSCE)**, n=3 (52,57,60)  **Canadian Centre for the Evaluation of Health Professionals Educated Abroad (CEHPEA) General Comprehensive Clinical Examination (CE1)**, n=1 (72)  **United States Medical Licensing Examination (USMLE)**, n=2 (62,65)  **UK General Medical Council’s (GMC) Professional and Linguistic Assessments Board (PLAB)**, n=2 (56,76)  **Assessment Procedure for IMGs in the Netherlands,** n=2 (49,51)  **Medical licensing examinations in Finland**, n=1 (67),  **Medical licensing examination in Sweden**, n=1 (53),  **Medical licensing examination in Germany**, n=1 (80)  **Unique National Exam of Medical Knowledge (EUNACOM) Chile**, n=1 (85).  **Postgraduate examinations**  **Royal College of General Practitioners Clinical Skills Assessment (RCGP CSA)**, n= 4 (47,63,66,73)  **Royal College of General Practitioners Workplace Based Assessment**, n=1 (75)  **Royal Australia and New Zealand College of Psychiatrists (RANZCP) fellowship examination**, n=2 (77,82)  **Undifferentiated UK Royal College examinations**, n=3 (78,79,83)  **Undifferentiated Australian Royal College examinations**, n=2 (3,64)  **Royal College of Paediatrics and Child Health (RCPCH) membership examination**, n=1 (70)  **Fellowship of the Royal College of Radiology (RCR) in Clinical Oncology**, n=1, (35)  **Australia and New Zealand College of Anaesthetists (ANZCA) fellowship examination**, n=1 (50)  **Fellowship of Royal Australian College of Surgery**, n=1 (54)  **US based Clinical Skills Verification Process (CSVP) postgraduate assessment for psychiat**ry, n=1 (74)  **Simulated examinations**, n=2  **RCGP CSA mock test run by Dorset Deanery in the UK**, n=1 (69)  **Simulated Surgery Examination of the GP Induction and Refresher** scheme which assesses the skills of EU GPs coming to work in the UK before they begin a period of supervised practice, n=1 (86) |
| **Specialty** | **General Practice (GP)**, n=7 (47,63,66,69,73,75,86)  **Psychiatry**, n=4 (56,74,77,82)  **Anaesthesiology**, n=1 (50)  **Oncology**, n=1 (35)  **Paediatrics**, n=1(70)  **Surgery**, n=1 (54)  **Mixed**, n= 12 [Including Anaesthesiology & Pain Medicine, Emergency Medicine, GP, Haematology, Internal Medicine, Laboratory medicine, Neurology, Obstetrics and Gynaecology, Oncology, Paediatrics, Psychiatry, Public Health & Preventive Medicine, Psychiatry, and Surgery] (3,52,55,64,67,71,72,76,78,79,81,83) |
