# Supplementary material for: International medical graduates’ experiences of clinical competency assessment in postgraduate and licensing examinations: A scoping review
Source: PLoS One. 2026 Apr 30;21(4):e0338282. doi: 10.1371/journal.pone.0338282 (PMC13132449; doi:10.1371/journal.pone.0338282)
Supplement: S5 Appendix — (DOCX) [file pone.0338282.s005.docx]

### S5: Appendix 5: Findings: Categories and sub-categories

| **Primary Category** | **Sub-category** | **#** | **Issues** | **References** |
| --- | --- | --- | --- | --- |
| Internal and personal factors | Practicalities | 6 | Family responsibilities preventing study | (Healey et al 2025; Higgins et al., 2010; Huijskens et al., 2010; Moneypenny, 2018; Sturesson et al., 2020; Webster & Ellison, 2010). |
|  |  | 11 | Financial problems | (Australian Medical Council, 2024; Ayonrinde, 2016; Bourgeault et al., 2010; Healey et al 2025; Huijskens et al., 2010; McGrath et al., 2012; Moneypenny, 2018; Parvathy et al 2021; Postmes & Rolim Medeiros, 2023; Rashid et al., 2023; Sood, 2019). |
|  | Communication challenges | 25 | Communication causing problem with exams | (Bourgeault et al., 2010; Cunningham et al., 2020; Harris & Guillemin, 2020; Heist & Torok, 2020; Huijskens et al., 2010; Iyizoba-Ebozue, Fatimilehin, Kayani, et al., 2024; Jamieson & Browne, 2011; Kuusio et al., 2014; Leung et al., 2020; Low et al., 2013; Mbeledogu et al., 2022; Moneypenny, 2018; Nair et al., 2015a; Peters, 2013; Postmes & Rolim Medeiros, 2023; Ragg et al., 2015; Rao et al., 2012; Remedios et al., 2010; Siriwardena et al., 2024; Slowther et al., 2012; Sturesson et al., 2020; Tipton, 2011; Vamos & Watson, 2009; Woolf, Rich, Viney, Needleman, et al., 2016; Woolf, Rich, Viney, Rigby, et al., 2016) |
|  | Impact on wellbeing | 12 | Worry about failing exam | (Australian Medical Council, 2024; Ayonrinde, 2016; Bourgeault et al., 2010; Harris & Guillemin, 2020; Iyizoba-Ebozue, Fatimilehin, Kayani, et al., 2024; Moneypenny, 2018; Ragg et al., 2015; Rashid et al., 2023; Sturesson et al., 2020; Tipton, 2011; Woolf, Rich, Viney, Needleman, et al., 2016; Woolf, Rich, Viney, Rigby, et al., 2016) |
|  |  | 6 | Awareness of differential attainment | (Australian Medical Council, 2024; Jamieson & Browne, 2011; Ragg et al., 2015; Sturesson et al., 2020; Woolf, Rich, Viney, Needleman, et al., 2016; Woolf, Rich, Viney, Rigby, et al., 2016). |
|  |  | 3 | Crisis of confidence after failure | (Iyizoba-Ebozue, Fatimilehin, Kayani, et al., 2024; Jamieson & Browne, 2011; Woolf, Rich, Viney, Rigby, et al., 2016). |
|  |  | 2 | Worry visa status or deportation | (Rao et al., 2012; Tipton, 2011). |
|  |  | 16 | Anxiety, depression, hopelessness and sleep disturbance | (Australian Medical Council, 2024; Bourgeault et al., 2010; Cunningham et al., 2020; Healey et al 2025; Healey et al 2025a; Loss et al., 2020; Postmes & Rolim Medeiros, 2023; Ragg et al., 2015; Rashid et al., 2023; Slowther et al., 2012; Sood, 2019; Sturesson et al., 2020; Tipton, 2011; Vamos & Watson, 2009; Woolf, Rich, Viney, Needleman, et al., 2016; Woolf, Rich, Viney, Rigby, et al., 2016). |
|  |  | 6 | Anger and frustration | (Australian Medical Council, 2024; Bourgeault et al., 2010; Peters, 2013; Postmes & Rolim Medeiros, 2023; Sood, 2019; Sturesson et al., 2020). |
| External and social factors | Employment challenges | 5 | Unable to get work experience or observership | (Kuusio et al., 2014; Loss et al., 2020; Peters, 2013; Postmes & Rolim Medeiros, 2023; Sood, 2019) |
|  |  | 6 | Length of time away from clinical work | (Australian Medical Council, 2024; Bourgeault et al., 2010; Healey et al 2025; Postmes & Rolim Medeiros, 2023; Sturesson et al., 2020; Tipton, 2011). |
|  |  | 7 | Those in medical jobs found that heavy workload impeded time to study | (Heist & Torok, 2020; Higgins et al., 2010; Mbeledogu et al., 2022; Siriwardena et al., 2024; Vamos & Watson, 2009; Webster & Ellison, 2010; Woolf, Rich, Viney, Rigby, et al., 2016) |
|  |  | 1 | Unaccredited service posts | (Australian Medical Council, 2024). |
|  |  | 9 | Prior training not recognised | (Australian Medical Council, 2024; Harris & Guillemin, 2020; Huthwaite et al., 2012; Legido-Quigley et al., 2015; Leung et al., 2020; Peters, 2013; Postmes & Rolim Medeiros, 2023; Sood, 2019; Vamos & Watson, 2009) |
|  |  | 6 | Fewer work or training opportunities because of IMG status | (Iyizoba-Ebozue, Fatimilehin, Kayani, et al., 2024; Peters, 2013; Rashid et al., 2023; Sood, 2019; Webster & Ellison, 2010; Woolf, Rich, Viney, Needleman, et al., 2016; Woolf, Rich, Viney, Rigby, et al., 2016) |
|  | Role of relationships | 14 | Support from Trainer and colleagues | (Heist & Torok, 2020; Iyizoba-Ebozue, Fatimilehin, Kayani, et al., 2024; Jamieson & Browne, 2011; Low et al., 2013; Mbeledogu et al., 2022; Nair et al., 2015a; Parvathy et al, 2021; Ragg et al., 2015; Slowther et al., 2012; Sood, 2019; Sturesson et al., 2020; Webster & Ellison, 2010; Woolf, Rich, Viney, Needleman, et al., 2016; Woolf, Rich, Viney, Rigby, et al., 2016). |
|  |  | 6 | Peer study groups | (Hodge, 2024; Jamieson & Browne, 2011; Low et al., 2013; McGrath et al., 2012; Ragg et al., 2015; Tipton, 2011) |
|  |  | 11 | Preparing with other IMGs | (Cunningham et al., 2020; Harris & Guillemin, 2020; Heist & Torok, 2020; Iyizoba-Ebozue, Fatimilehin, Kayani, et al., 2024; Loss et al., 2020; McGrath et al., 2012; Moneypenny, 2018; Ragg et al., 2015; D. R. Terry et al., 2014; Woolf, Rich, Viney, Needleman, et al., 2016; Woolf, Rich, Viney, Rigby, et al., 2016). |
|  |  | 3 | Preparing with local graduates | (Ragg et al., 2015; Sturesson et al., 2020; Woolf, Rich, Viney, Rigby, et al., 2016) |
|  |  | 3 | Community integration | (Ragg et al., 2015; Sturesson et al., 2020; Woolf, Rich, Viney, Needleman, et al., 2016). |
|  |  | 11 | Social and geographical isolation | (Higgins et al., 2010; Hodge, 2024; Iyizoba-Ebozue, Fatimilehin, O’Reilly, et al., 2024; Moneypenny, 2018; Nair et al., 2015b; Postmes & Rolim Medeiros, 2023; Ragg et al., 2015; Sturesson et al., 2020; Tipton, 2011; Webster & Ellison, 2010; Woolf, Rich, Viney, Needleman, et al., 2016). |
|  | Different models of practice | 8 | Different models of practice in home countries | (Jamieson & Browne, 2011; Peters, 2013; Postmes & Rolim Medeiros, 2023; Ragg et al., 2015; Rashid et al., 2023; Sood, 2019; Tipton, 2011; Woolf, Rich, Viney, Rigby, et al., 2016). |
|  |  | 5 | Unfamiliar with patient centred care | (Cunningham et al., 2020; Harris & Guillemin, 2020; Jamieson & Browne, 2011; Legido-Quigley et al., 2015; Slowther et al., 2012). |
|  |  | 6 | Value of bridging programmes | (Australian Medical Council, 2024; Bourgeault et al., 2010; Jamieson & Browne, 2011; McGrath et al., 2012; Sood, 2019; Woolf, Rich, Viney, Rigby, et al., 2016). |
|  |  | 13 | Difference in cultural practice causing a problem in exams | (Ayonrinde, 2016; Bourgeault et al., 2010; Healey et al, 2025; Iyizoba-Ebozue, Fatimilehin, Kayani, et al., 2024; Moneypenny, 2018; Nair et al., 2015a; Peters, 2013; Postmes & Rolim Medeiros, 2023; Ragg et al., 2015; Rao et al., 2012; Slowther et al., 2012; Tipton, 2011; Woolf, Rich, Viney, Rigby, et al., 2016). |
| Institutional factors | Teaching and Learning and Assessments | 10 | Methods of assessment were different from what they were used to | (Kunakov & Bozzo, 2015; Peters, 2013; Rao et al., 2012; Rashid et al., 2023; Remedios et al., 2010; Siriwardena et al., 2024; Sood, 2019; Tipton, 2011; Webster & Ellison, 2010; Woolf, Rich, Viney, Rigby, et al., 2016) |
|  |  | 16 | Difficult to find information about the assessment | (Australian Medical Council, 2024; Healey et al, 2025; Heist & Torok, 2020; Higgins et al., 2010; Huijskens et al., 2010; Huthwaite et al., 2012; Khan et al., 2019; Kuusio et al., 2014; Loss et al., 2020; McGrath et al., 2012; Moneypenny, 2018; Peters, 2013; Sood, 2019; D. R. Terry et al., 2014; Vamos & Watson, 2009; Webster & Ellison, 2010) |
|  |  | 7 | Examinations were unrealistic and not reflective of practice | (Australian Medical Council, 2024; Huthwaite et al., 2012; Jamieson & Browne, 2011; Khan et al., 2019; Legido-Quigley et al., 2015; Sturesson et al., 2020; Woolf, Rich, Viney, Needleman, et al., 2016). |
|  |  | 3 | IMGs behaved differently in the examination to increase chances of passing | (Harris & Guillemin, 2020; Woolf, Rich, Viney, Needleman, et al., 2016; Woolf, Rich, Viney, Rigby, et al., 2016). |
|  |  | 3 | WBA considered more realistic | (Nair et al., 2012,; Parvathy et al, 2021; Siriwardena et al., 2024). |
|  | Bureaucracy | 8 | Bureaucratic hurdles | (Australian Medical Council, 2024; Bourgeault et al., 2010; Healey et al, 2025; Healey et al, 2025a; Kuusio et al., 2014; Loss et al., 2020; Sood, 2019; D. R. Terry et al., 2014) |
|  |  | 5 | Refugees | (Bourgeault et al., 2010; Huijskens et al., 2010; Loss et al., 2020; Postmes & Rolim Medeiros, 2023; Slowther et al., 2012) |
|  | Feedback | 4 | Insufficient feedback received after failing examinations | Australian Medical Council, 2024; Jamieson et al, 2011; Peters, 2013; Webster et al, 2010 |
|  |  | 4 | IMGs were more satisfied with feedback received from WBA | Nair et al, 2012; Nair et al 2015; Terry et al, 2020; Parvathy et al 2021 |
|  | Perception of fairness | 8 | IMGs feeling discriminated against | (Australian Medical Council, 2024; Healey et al 2025; Healey et al 2025a; Iyizoba-Ebozue et al, 2024; Moneypenny, 2018; Siriwardena et al., 2024; Sood, 2019; Woolf, Rich, Viney, Needleman, et al., 2016; Woolf, Rich, Viney, Rigby, et al., 2016). |
|  |  | 5 | Accent might be a cause of bias | (Healey et al 2025; Iyizoba-Ebozue et al., 2024; Jamieson & Browne, 2011; Moneypenny, 2018; Woolf, Rich, Viney, Needleman, et al., 2016). |
|  |  | 14 | Test requirements considered unfair | (Australian Medical Council, 2024; Bourgeault et al., 2010; Harris & Guillemin, 2020; Healey et al, 2025; Healey et al 2025a; Kuusio et al., 2014; Legido-Quigley et al., 2015; Loss et al., 2020; Moneypenny, 2018; Peters, 2013; Siriwardena et al., 2024; Sood, 2019; Sturesson et al., 2020; Tipton, 2011). |
|  |  | 4 | Other forms of assessment fairer than OSCEs | (Woolf, Rich, Viney, Rigby, et al., 2016; Khan et al., 2019; Kunakov & Bozzo, 2015; Siriwardena et al., 2024). |
|  |  | 7 | Examiner preferences could cause failure | (Australian Medical Council, 2024; Healey et al 2025a; Nair et al., 2015a; Siriwardena et al., 2024; Sturesson et al., 2020; D. Terry et al., 2020; Woolf, Rich, Viney, Rigby, et al., 2016). |
|  |  | 3 | Effect of knowing the examiner in WBA | (Nair et al., 2012; D. Terry et al., 2020) - Positive effect; (Siriwardena et al., 2024) - negative effect |
|  |  | 1 | IMGs unfamiliar with the role of SPs | (Tipton, 2011) |
