# Supplementary material for: International medical graduates’ experiences of clinical competency assessment in postgraduate and licensing examinations: A scoping review
Source: PLoS One. 2026 Apr 30;21(4):e0338282. doi: 10.1371/journal.pone.0338282 (PMC13132449; doi:10.1371/journal.pone.0338282)
Supplement: Attachment — Submitted filename: Response to Reviewers.docx [file pone.0338282.s006.docx]

Dr Helen Hynes,

College of Medicine and Health

School of Medicine,

Brookfield health Science Complex,

University College Cork,

College Road, Cork, Ireland

April 6th, 2026

Dr Kento Sonoda, MD
Academic Editor

PLOS One.

Dear Dr Sonoda and Reviewers,

Many thanks for reviewing our article – “International medical graduates’ experiences of clinical competency assessment in postgraduate and licensing examinations: A scoping review” [PONE-D-25-62031] and for the opportunity to resubmit with revisions.

We have considered each of the points made by the reviewers. We are grateful for the suggestions and have made the following amendments in response to same.

Reviewer #1

| **Section** | **Suggested revision** | **Action taken** |
| --- | --- | --- |
| Abstract | Line 52: Revise ‘conclusions’ to ‘conclusion’ | Revised as suggested |
| Methodology | Line 135: The process followed Arksey & Omalley’s Framework. However, line 32 under the abstract indicated that the Joana Briggs Institute was employed in this scoping review. Please revise this anomaly in the abstract and under the methodology section. I suggest the authors state this clearly, as it forms the heart of this scoping review. | Thank you. This has been clarified by removing the reference to using Joanna Briggs methodology - see line 32 of the abstract. |
| Line 166: Study selection. | • Please, the content under this section does not indicate the number of publications identified from the initial search. After deduplication (how many duplicates were removed), how many were removed after abstracts and titles were screened? What were the reasons for their removal? These review comments are relevant mainly because the PRISMA Flow Diagram was illegible due to poor image quality. I suggest that the authors revise this section, as it shows the validity and reliability of the scoping review.  • I suggest that the authors provide a table showing the published materials considered after removing duplicates, clearly indicating those included and excluded, as well as the reasons for exclusion. | The Prisma flow diagram has been re-saved. It is now legible and shows the details required. |
| Line 183: | A pilot test was conducted to trial the inclusion criteria. | A description has been added of how this took place. |
| Line 150: Identifying relevant sources for inclusion: | In the abstract, the authors indicated that there was no language limitation. However, the methodology section did not mention this criterion. I suggest that the authors revise this section to indicate whether some peer-reviewed articles (number specified) were identified in other languages (language specified) and the decision made regarding such articles. This information needs to be included in the ‘description of the sources.’ | This information has now been added to the section on "description of the sources". |
| Line 432-425: | Please, revise for clarity. | Thank you. This has been revised for clarity |
| Line 471: | What does ‘this’ mean in that quote/extract? | "This” refers to OSCE type assessment - this has now been clarified in the text. |
| Line 510: | Revise the quote for clarity. Consider ‘Where can I get my birth certificate?’ | Thank you. Edited as advised |
| Line 591: | Revise (Tipton, 2011) to be consistent with the preferred referencing style of the journal. | Edited. Thank you for spotting this. |
| Line 622: | Please, do not start a sentence with a number (44), revise to Forty-four (44) | Edited. Thank you. |
| Line 885: | I suggest the authors check this reference to confirm the presence of the dot between physician and Le (Physician . Le) | Amended. Thank you. |
| Supporting Information | S1: Figure 1, Scoping Review PRISMA Flow Diagram is blurred. Hence, difficult to appreciate. I suggest that the authors upload a clearer figure. | The Prisma flow diagram has been re-saved. It is now legible and shows the details required. |
| S4: Description of Sources | I suggest the authors used descriptive statistics to describe the sources under the following headings:  • Year  • Country of publication  • Type of study  • Examination  • Specialty | The section on description of the sources has been amended to include descriptive statistics as suggested. |

Reviewer #2

| **Section** | **Suggested revision** | **Action taken** |
| --- | --- | --- |
| Data extraction process | The authors note that while the data extraction tool was piloted by three reviewers working in pairs, the remaining extraction was carried out solely by the principal investigator (HH). For a scoping review aiming for methodological rigour, it would strengthen the paper if the authors could clarify what steps were taken to verify the accuracy and consistency of the single-reviewer extraction, or acknowledge this more explicitly as a limitation. | Thank you. The remaining data extraction was carried out by the principal investigator (HH) and was reviewed for accuracy and consistency by the other members of the research team. This has now been clarified in the text. Please see lines 208 -209 in the section on Data Extraction in the Revised Manuscript with tracked changes |

Thank you sincerely for your consideration of this manuscript.

Warm Regards,

Dr Helen Hynes, MB, BCh, BAO, MICGP, MSc (Med Ed)

Senior Lecturer in Medical Education,

University College Cork, College Road, Cork, Ireland
